# A survey of deepwater horizon (DWH) oil-degrading bacteria from the Eastern oyster biome and its surrounding environment

**DOI:** 10.3389/fmicb.2014.00149

**Published:** 2014-04-09

**Authors:** Jesse C. Thomas, Denis Wafula, Ashvini Chauhan, Stefan J. Green, Richard Gragg, Charles Jagoe

**Affiliations:** ^1^Environmental Biotechnology Laboratory, School of the Environment, Florida Agricultural and Mechanical UniversityTallahassee, FL, USA; ^2^DNA Services Facility, University of Illinois at ChicagoChicago, IL, USA; ^3^Department of Biological Sciences, University of Illinois at ChicagoChicago, IL, USA; ^4^NOAA Environmental Cooperative Science Center, School of the Environment, Florida Agricultural and Mechanical UniversityTallahassee, FL, USA

**Keywords:** deepwater horizon oil spill, oyster, bacteria, biodegradation, hydrocarbon

## Abstract

The deepwater horizon (DWH) accident led to the release of an estimated 794,936,474 L of crude oil into the northern Gulf of Mexico over an 85 day period in 2010, resulting in the contamination of the Gulf of Mexico waters, sediments, permeable beach sands, coastal wetlands, and marine life. This study examines the potential response of the Eastern oyster’s microbiome to hydrocarbon contamination and compares it with the bacterial community responses observed from the overlaying water column (WC) and the oyster bed sediments. For this purpose, microcosms seeded with DWH crude oil were established and inoculated separately with oyster tissue (OT), mantle fluid (MF), overlaying WC, and sediments (S) collected from Apalachicola Bay, FL, USA. Shifts in the microbial community structure in the amended microcosms was monitored over a 3-month period using automated ribosomal intergenic spacer region analysis, which showed that the microbiome of the OT and MF were more similar to the sediment communities than those present in the overlaying WC. This pattern remained largely consistent, regardless of the concentration of crude oil or the enrichment period. Additionally, 72 oil-degrading bacteria were isolated from the microcosms containing OT, MF, WC, and S and identified using 16S ribosomal RNA gene sequencing and compared by principal component analysis, which clearly showed that the WC isolates were different to those identified from the sediment. Conversely, the OT and MF isolates clustered together; a strong indication that the oyster microbiome is uniquely structured relative to its surrounding environment. When selected isolates from the OT, MF, WC, and S were assessed for their oil-degrading potential, we found that the DWH oil was biodegraded between 12 and 42%, under the existing conditions.

## INTRODUCTION

Since the start of the second industrial revolution, the use of fossil fuels including petroleum hydrocarbons has increased dramatically (Mojib et al., 2011). The world’s total oil consumption is expected to rise to over 96.1 million barrels/day by 2015 and up to 113.3 million barrels/day by 2030 [United States Energy Information Administration (US-EIA), 2011a]. As of 2010, the United States alone consumed 19.15 million barrels/day of refined petroleum products and biofuels; which is approximately 22% of the world’s total petroleum consumption (United States Energy Information Administration (US-EIA), 2011b). However, despite stimulating growth in areas such as agriculture and transportation, petroleum fuels are a major source of environmental pollution by way of accidental spills, particularly in estuarine and marine ecosystems (Paine et al., 1996; Newman and Unger, 2003).

When coastal ecosystems are impacted by contaminants, important functions (e.g., fishery production, biofiltration) may be affected (Livingston, 1984; Nixon, 1995). Oil spills such as the 1989 Exxon Valdez accident in Alaska and more recently, the deepwater horizon (DWH) spill illustrate how oil hydrocarbons can cause acute (e.g., lethality) and chronic (e.g., reduced growth and foraging success, reduced fecundity, increased levels of deformities, and abnormal social behavior) impacts to coastal ecosystem services (Peterson, 1991, 2001; Peterson et al., 2003; Hazen et al., 2010; Kostka et al., 2011; Henkel et al., 2012; Whitehead et al., 2012). Assessing impacts of oil spills requires a comprehensive understanding of ecosystem services, including the water filtration capacity and nursery areas that are typically provided by oyster reef habitats (Peterson and Heck, 1999; French McCay et al., 2003).

As of 2000, estuaries in the Gulf of Mexico region contributed 83% of the total Eastern oysters (*Crassostrea virginica*) harvested in the United States (U.S. National Marine Fisheries Service^1^). As a keystone species in the Gulf, oysters and their reef assemblages serve as critical habitat for commercially important seafood, while also performing critical ecosystem services such as water filtration and sequestration of excess nitrogen (Raj, 2008; Roosi-Snook et al., 2010; Piehler and Smyth, 2011; Grabowski et al., 2012). The value of this habitat, both ecologically and economically, however, may be greatly reduced by pollution, including accidental oil spills.

It should however, be noted that the Gulf of Mexico waters are continuously exposed to a background level of hydrocarbons vented by numerous hydrocarbon seeps, which emit hydrocarbon gas into the overlaying water column (WC) as bubble plumes which are often coated with a thin layer of oil (MacDonald et al., 2002; Leifer and MacDonald, 2003; Solomon et al., 2009). According to some estimates, seepage in the Gulf of Mexico, from over 200 active seeps, contributes oil at a high rate of 0.4–1.1 × 10^8^ L/year (Mitchell et al., 1999). As a corollary, this “natural” oil likely primes the environment continuously, by selectively enriching native microorganisms with the ability to metabolize and mineralize some of this oil. Therefore, it is very likely that a suite of native hydrocarbon-degrading bacteria exist in different niches of the Gulf of Mexico, and as reported previously responded rapidly to the large volume of oil released by the DWH spill (Hazen et al., 2010; Redmond and Valentine, 2012; Dubinsky et al., 2013; Gutierrez et al., 2013).

A significant body of information exists on the pathways and the nature of oil-degrading microbes from a variety of diverse environments, including soils and aquatic ecosystems (Margesin and Schinner, 2001; Aitken et al., 2004; Hamamura et al., 2005; Hazen et al., 2010; Kostka et al., 2011), but virtually nothing is known on the nature and ability of microbes that can degrade oil from the oyster reefs or within the oyster itself. In this report, we have investigated the propensity of a largely understudied ecosystem- the Eastern oyster (*Crassostrea virginica*) microbiome – to degrade the Gulf crude oil. Previous studies have shown that the oyster biome consists of a diverse collection of heterotrophic bacteria that differ from the surrounding seawater in both species composition and abundance (Kueh and Chan, 1985; Pujalte et al., 1999; Campbell and Wright, 2003). Moreover, oyster-associated bacteria often outnumber the bacterial populations present in the WC by several orders of magnitude (Kelly and Dinuzzo, 1985; Mahasneh and Al-Sayed, 1997), most likely due to the oyster’s filter-feeding behavior (Galtsoff, 1964; Haven and Morales-Alamo, 1970; Jorgensen, 1990). Romero et al. (2002) propose two kinds of bacteria found within oysters- autochthonous, which are relatively permanent and remain associated with the oysters and allochthonous, which are passing through the oysters due to the filter-feeding processes. The autochthonous groups are even known to have a strong symbiotic relationship with their bivalve host.

In fact, Colwell and Liston (1960) first suggested the existence of a defined commensal flora in oysters. More recently, the findings of Zurel et al. (2011) have shown that a conserved seasonal association between the Chama-associated oceanospirillales group (CAOG) of bacteria and oysters likely represents a symbiotic association. Trabal et al. (2012) reported a symbiotic host-bacteria relationship during different growth phases of two oyster species- *Crassostrea gigas* and *Crassostrea corteziensis*. Such symbionts may assist in the digestion processes, as has been demonstrated in the larvae of *Crassostrea gigas* (Prieur et al., 1990), and may also supply the bivalve host with vitamins and amino acids that serve as growth factors- as shown in the Pacific vesicomyid clam- *Calyptogena magnifica* found at cold seeps (Newton et al., 2007). Moreover, certain symbiotic bacteria can even protect their host from pathogens by either producing antimicrobial agents, or by growing in high densities that prevents colonization by other strains (Pujalte et al., 1999). More recently, King et al. (2012) used pyrosequencing to reveal substantial differences between stomach and gut microbiomes of oysters from Lake Caillou in Louisiana. These authors found that bacteria belonging to *Chloroflexi*, *Mollicutes*, *Planctomycetes,* and *Spartobacteria* likely comprise a major core of the oyster stomach microbiome, whereas, *Chloroflexi*, *Firmicutes*, *α-proteobacteria*, and *Verrucomicrobia* were more abundant in the gut.

Despite the existing information on the nature of oyster-associated microbial communities, not much is known on their ability to degrade oil hydrocarbons. Therefore, the purpose of this study was to not only to assess the oyster microbiome, which in itself necessitates further studies because it is a largely understudied microhabitat, but also to enrich, isolate and compare the oil biodegradation efficacy of oyster-associated bacteria.

## MATERIALS AND METHODS

### SITE DESCRIPTION AND SAMPLE COLLECTION

This study was conducted on oysters, water, and sediment (S) samples collected from Dry Bar, the most productive bar, in Apalachicola Bay, Florida (29° 40.474N, 085°03.497W). Apalachicola Bay is a relatively pristine estuary, well mixed by freshwater from the Apalachicola-Chattahoochee-Flint (ACF) river system and oceanic Gulf tides (Chauhan et al., 2009). The bay produces 90% of Florida oysters, the third highest catch of shrimp, a rich supply of brown shrimp, scallops, and blue crabs (Livingston, 1984).

Samples for this study were collected on June 14, 2011. Before collecting the environmental samples, physiochemical parameters were measured with a YSI probe, which included salinity (26.5 parts per thousand, ppt), dissolved oxygen (7.2 mg/L), conductivity (45.37 mS), and temperature (30.1°C). Oysters were collected using a tong, culled, and 20 adult oysters, of approximately the same size were collected. Additionally, 1 L of water from directly above the oyster bed was collected in a sterile bottle, and approximately 10 g of sediment was collected into a sterile container from below the oyster beds using a sediment grab sampler. All samples were stored on ice and transported to Florida Agricultural and MechanicalUniversity for further processing on the same day samples were collected.

### ENRICHMENT OF OIL-DEGRADING BACTERIA

Oysters were carefully culled and rinsed using sterile 0.85% NaCl to remove debris and shell biofilm. Prior to collection of oyster tissues (OT), mantle fluid (MF) from each oyster was aseptically collected by opening each oyster from the hinge side and aspirating the fluid from the mantle cavity by using sterile syringes fitted with 21 gage needles. By MF, we are referring to the fluid accumulated within the mantle cavity (Pekkarinen, 1997; Sadok et al., 1999). In other words, the liquid within the mantle cavity enclosed by the valves that bathes the internal tissues including the gills. This is also sometimes called the mantle cavity fluid. Each oyster was then carefully shucked using sterile knives, and OT was then homogenized in pre-sterilized blenders and collected into sterile Falcon tubes (BD Biosciences, San Jose, CA, USA).

Enrichment of oil-degrading bacteria was performed in Bushnell-Haas (BH) medium containing: MgSO_4_ 0.2 g/L, CaCl_2_ 0.02 g/L, KH_2_PO_4_ 1 g/L, (NH_4_)_3_PO_4_ 1g/L, KNO_3_ 1 g/L, and FeCl_3_ 0.05 g/L, pH 7.1 (Bushnell and Haas, 1941) with a salinity of 3.2 ppt. Even though the measured salinity of the oyster sampling site was higher than that of our enrichment microcosms, the typical salinity of the sampled site is highly dynamic ranging from 3 to 33 ppt, depending on the season and freshwater input from the riverine flows (Livingston et al., 2000). Enrichments contained 100 mL of media and 1% OT, MF, S, or water and supplemented with 0.1% (v/v) or 1% (v/v) of filter-sterilized crude oil that was obtained directly from the source of the Deep Horizon spill site. The oil was provided as the sole source of carbon and energy, and a full characterization of the oil has been reported elsewhere (Kostka et al., 2011). After 1 month of incubation on a rotary shaker at 30°C, 1 mL of enriched sample was transferred into fresh BH media containing 0.5% crude oil and incubation was continued for another month. After the second enrichment, 0.4 mL of sample was serially diluted and plated on BH agar media. Isolated colonies from different environments were streaked onto BH agar media and exposed to oil vapors using filters soaked in 500 μL of crude oil, according to the method of Kleinheinz and Bagley (1997). Those colonies that grew on BH media exposed to oil vapors were re-streaked onto fresh oil-amended liquid media and further purified by a second round on BH-vapor plates.

### NUCLEIC ACID EXTRACTION AND AUTOMATED RIBOSOMAL INTERGENIC SPACER REGION ANALYSIS

**FIGURE 1:**
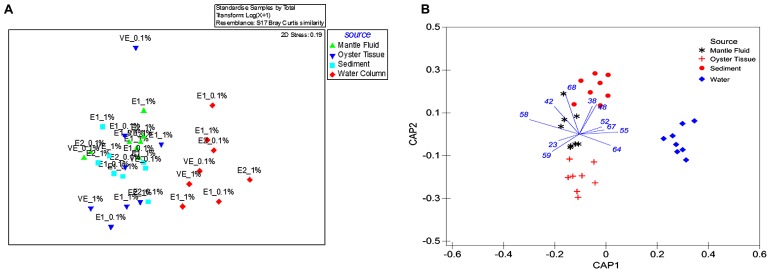
**Shown are (A) non-metric multidimensional scaling plot (NMDS) of ARISA data obtained from oyster tissue (blue triangle), mantle fluid (green triangle), water column (red diamond), and sediments (turquoise square) with sample source as the grouping factor.** The bacterial communities from enrichment stages are shown as E1 (first enrichment), E2 (second enrichment) and VE (vapor enrichment); **(B)** CAP on the ITS region gene sequences obtained by ARISA with sample source as the grouping factor. 1 and 0.1% denotes the percentage of crude oil amended in each of the enrichment microcosms.

Automated ribosomal intergenic spacer region analysis (ARISA) that employs the bacterial 16S to 23S rRNA intergenic spacer region was used to assess changes in bacterial community structure over the duration of oil-amended enrichments. From the first set (**Figure 1A**) and second set (**Figure 1B**) of oil enrichments established in liquid media, approximately 80 mL of media was collected after filtered through 0.2 μm filter followed by DNA extraction of the filtered biomass using the MOBIO PowerSoil DNA Isolation Kit according to the manufacturer’s instructions (MOBIO Laboratories, Carlsbad, CA, USA). The biomass that grew on BH-vapor plates after the second round of enrichment was scraped from the agar surface and resuspended in 10 mL of 0.85% NaCl and centrifuged at 10,000 *g* for 5 min. The supernatant was decanted and DNA was isolated from the pellet using the MOBIO PowerSoil DNA Isolation Kit. Concentration and quality of DNA was determined using a micro-volume spectrophotometer (NanoDrop Technologies, Wilmington, DE, USA). This constituted the hydrocarbon vapor enrichment (VE) phase.

The fluorescently tagged primers ITSF (5′-GTCGTAACAAGG-TAGCCGTA-3′) and ITSReub (5′-GCCAAGGCATCCACC-3′) were used to amplify bacterial ITS regions (Cardinale et al., 2004). PCR reactions contained 25 μL of GoTaq Colorless Master Mix (Promega, Madison, WI, USA), 2 μL of each primer previously diluted to a concentration of 5 pmol, molecular grade water and 50–100 ng of genomic DNA. Thermal cycling conditions consisted of an initial denaturation of 3 min at 94°C, followed by 30 cycles of 45 s at 94°C, 55°C for 1 min, and a final elongation step of 10 min at 72°C. Resulting PCR products were purified using UltraClean PCR clean-up kit according to the manufacturer’s protocol (MoBio Laboratories, Carlsbad, CA, USA), quantified, and diluted as stated above. Samples were analyzed by the DNA Services Facility, University of Illinois at Chicago, using an ABI 3730xl capillary electrophoresis system (Life Technologies, Inc., Carlsbad, CA, USA).

After manually examining the electropherogram data and images, background noise thresholds were set to 10 fluorescent units. The resultant peak area data matrix was exported into Microsoft Excel^®^ where a custom script chose peaks that were between 100 and 1000 bp; all peaks within 5 bp were binned to reduce peak calling variation which can occur during sample analysis. Sizing error during ARISA increases with fragment length, and binning or other correction strategies such that any errors associated with run-to-run variability are minimized. Because the ARISA fragments from the samples analyzed in this study ranged approximately between 700 and 1000 bp, we kept the binning threshold at 5 bp (Brown et al., 2005). The data matrix was processed as follows: triplicate runs were averaged to produce a single peak area for each sample, imported into the Primer 6 software suite (version 6.1.10; PRIMER-E, Ivybridge, UK) and normalized using the log (X + 1) pre-treatment function. After transformation, a Bray-Curtis similarity matrix was generated and used as a basis for permutational multivariate analysis of variance (PERMANOVA). Additionally, the data were analyzed using a non-metric multidimensional scaling plot (NMDS), which was compared directly with canonical analysis of principal components (CAP).

### ISOLATION AND CHARACTERIZATION OF OIL-DEGRADING BACTERIA

From the oil-vapor enriched plates, six colonies from each environment (OT, MF, water, and S), having similar morphologies, were streaked on salt-water yeast extract (SWYE) agar plates consisting of: peptone 10 g/L, yeast extract 3 g/L in 70% artificial seawater, pH 7.1, agar 15 g/L. These isolates were then transferred onto fresh BH agar plates and exposed to crude oil vapors incubated in airtight glass desiccators held at 30°C. After 10 days, colonies that appeared on the crude oil vapor-exposed plates were picked and streaked onto fresh SWYE agar plates. A total of 118 single colonies were re-streaked onto BH agar plates and exposed to oil vapor again so as to obtain axenic isolates. Finally, pure bacterial isolates from the OTs, MF, WC, and S environments were streaked onto SWYE for regular maintenance. For long-term storage, isolates were stored in 15% glycerol, at -80°C.

A direct colony PCR method was used to amplify the 16S rRNA gene from 72 pure isolates obtained from the enrichments. For this, a single colony representing each of the isolated strain was picked using a sterile toothpick and resuspended in a premix of buffer, primers, and taq polymerase. Universal eubacterial primers 1492R and 27F were used for 16S rRNA gene sequencing and subsequent identification of the isolated bacteria as shown previously (Chauhan et al., 2009). Bacterial 16S rRNA gene sequences were then aligned using Greengenes^2^, aligned using MEGA ver. 6.0 and a phylogenetic tree was constructed using the neighbor-joining distance method with a bootstrap test of 500 replicates (Tamura et al., 2013). The evolutionary distances were computed using the Maximum Composite Likelihood method in the units of the number of base substitutions per site.

### NUCLEOTIDE SEQUENCE ACCESSION NUMBERS

The partial 16S rRNA gene sequences obtained in this study are available in GenBank under accession numbers of JX501343–JX501351.

### GROWTH KINETICS AND OIL BIODEGRADATION POTENTIAL OF REPRESENTATIVE ISOLATES

Nine isolates were chosen to evaluate growth and oil degradation efficiencies. These particular isolates were chosen because either (1) they were found only in a specific environment, as was the case with *Pseudomonas alcaligenes*, which was isolated only from the S, or (2) they were common between several environments, as was the case with *P. montelii* isolated from S, OT, and MF, respectively.

Briefly, single colonies of the selected isolates were inoculated into 5 mL SWYE media at 30°C overnight. Each of the cultures, in triplicate, was then centrifuged for 5 min at 5,000 × *g*, rinsed three times with 5 mL of BH media and OD_600_ was adjusted to <0.2 using BH media. A Bioscreen C honeycomb plate (Growth Curves USA, NJ, USA) was then inoculated with 350 μL of cell suspension containing 0.75% crude oil, and incubated at 30°C within the Bioscreen C system. To compare growth rates, tests were run without crude oil in BH media containing 0.75% glucose, and control ODs were subtracted from the tests to assess growth rate of each isolate. Bioscreen C was set to measure OD_450__-__580_ using the wide-band filter at 6 h intervals for 7 days and the doubling time of each isolate was calculated after the completion of the growth experiments, as shown previously (Moat and Foster, 1995).

To estimate the ability of each of the nine isolates to degrade crude oil, a gravimetric method was used (Kostka et al., 2011). Briefly, single colonies of the selected isolates were inoculated into 5 mL SWYE media at 30°C overnight. The cell cultures were centrifuged for 5 min at 5,000 × *g*. The cell pellet was rinsed three times with 5 mL of BH media until OD_600_ was approximately 0.2. Flasks containing 50 mL of BH media were inoculated with 1% (v/v) of actively growing culture, supplemented with 0.75% crude oil and incubated on a rotary shaker at 175 rpm, 30°C for 7 days; after which the residual oil was extracted twice using 50 mL of chloroform. In order to concentrate the remaining oil, the chloroform extract was transferred into pre-weighed glass vials and held in a water bath at 60°C. This caused the chloroform to evaporate leaving behind the residual oil, which was then compared to the initial weight of the amended oil. The average amount of oil lost in the controls (abiotic) was subtracted from the amount of oil lost from the flasks containing the isolates (biotic) to estimate the amount of oil biodegraded by the isolates.

## RESULTS AND DISCUSSION

### COMPARISON OF BACTERIAL COMMUNITY COMPOSITION IN OIL ENRICHED MICROCOSMS

Bacterial communities that could metabolize oil from OTs and MF were compared with those found in the surrounding environment- the overlaying WC and oyster bed S by ARISA of bacterial 16S rRNA genes using a NMDS (**Figure 1A**). The single sampling time performed for this study potentially limits extrapolation of our findings, but at the same time, provides a new perspective on the largely understudied oyster-associated microbiome and directions for future studies.

Despite that the initial comparison between the bacterial communities in the OT, MF, WC, and S was performed only after being enriched in crude oil for a month, we were still able to detect distinct differences. Specifically, the WC bacteria clustered separately and away from those present in the OTs, MF, and S, respectively (**Figure 1A**). This suggests that the microbiome of OT, MF and S were more similar to each other relative to the WC regardless of the period for which they were enriched in liquid media or exposed to VEs or the concentration of amended oil used in the microcosms (0.1 or 1%). Furthermore, although powerful for visualizing broad patterns, unconstrained ordination methods such as NMDS are known to mask certain patterns among groups dispersed in experimental data (Anderson and Willis, 2003; Anderson and Robinson, 2003). Therefore, a constrained ordination method – the canonical analysis of principal CAP analysis was used, and this demonstrated a significant clustering of bacterial communities selected for oil degradation based on the source environment (**Figure 1B**); this analysis mirrored the NMDS representation such that the WC bacterial communities clustered together and away from the OT microbiome. Furthermore, CAP demonstrated an 87.5% classification rate, and resulted in the discrimination of the MF bacteria which appeared closer to the OT than those that were present in the S.

**FIGURE 2:**
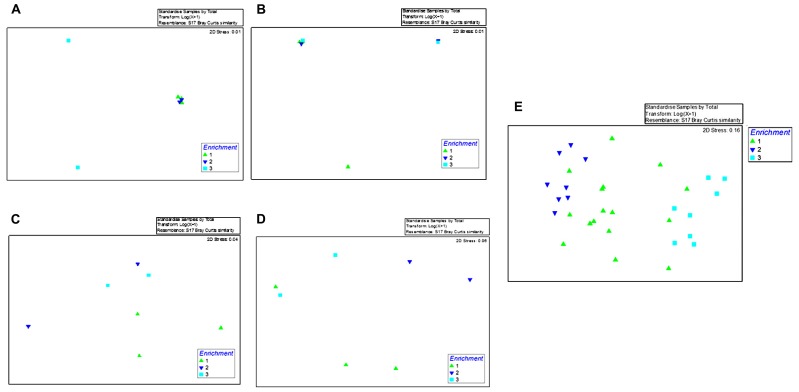
**Shown are the non-metric multidimensional scaling plot (NMDS) of ARISA data obtained from the first enrichment phase (E1; green triangle), second enrichment phase (E2; blue triangle), and the third enrichment phase (VE; turquoise square) with enrichment time as the grouping factor.** Shown in **(A–D)** are bacterial community profiles within the samples enriched with oyster tissue **(A)**, mantle fluid **(B)**, sediments **(C)**, and water **(D)**, respectively. **(E)** Shows pooled ARISA data from the oyster tissue, mantle fluid, sediments, and water over the course of this study.

Moreover, to assess whether the bacterial communities from each of the tested environments (OT, MF, S, and WC) shifted over the course of the enriched time in the presence of crude oil, we performed NMDS using the time of enrichment as the variable factor (E1 = 1; E2 = 2; and VV = 3) on each sampled environment separately (**Figures 2A–D**) or when pooled together regardless of their origin (**Figure 2E**). This analysis clearly showed that after 1 month of enrichment, communities were still relatively divergent (**Figures 2A–E**). Conversely, during the second and the third enrichment phases, the biodegradative communities became distinctly different suggesting that bacteria had become sufficiently enriched over the course of this study.

A pair wise comparison of data using PERMANOVA showed the same trend as shown by the NMDS analysis, i.e., the OT s (*P* = 0.037) and MF (*P* = 0.012) were more similar to S than the WC (*P* = 0.003 or less). Unexpectedly, MF and OTs displayed greater similarity with the S than with each other (*P* = 0.0093) and, this pattern of association was consistent, regardless of the concentration of crude oil or time of exposure (data not shown). These findings however, should be interpreted with the caveat that we did not assess the native bacterial communities in a “no-oil” spiked microcosm containing the OT, MF, water, and S, respectively. Despite this limitation in our experimental design, we do provide a preliminary baseline on the response of a largely understudied environment- the oyster microbiome and its response to the addition of oil hydrocarbons, upon which additional studies can be conducted.

### TAXONOMIC ANALYSIS OF THE OIL-DEGRADING ISOLATES

**FIGURE 3:**
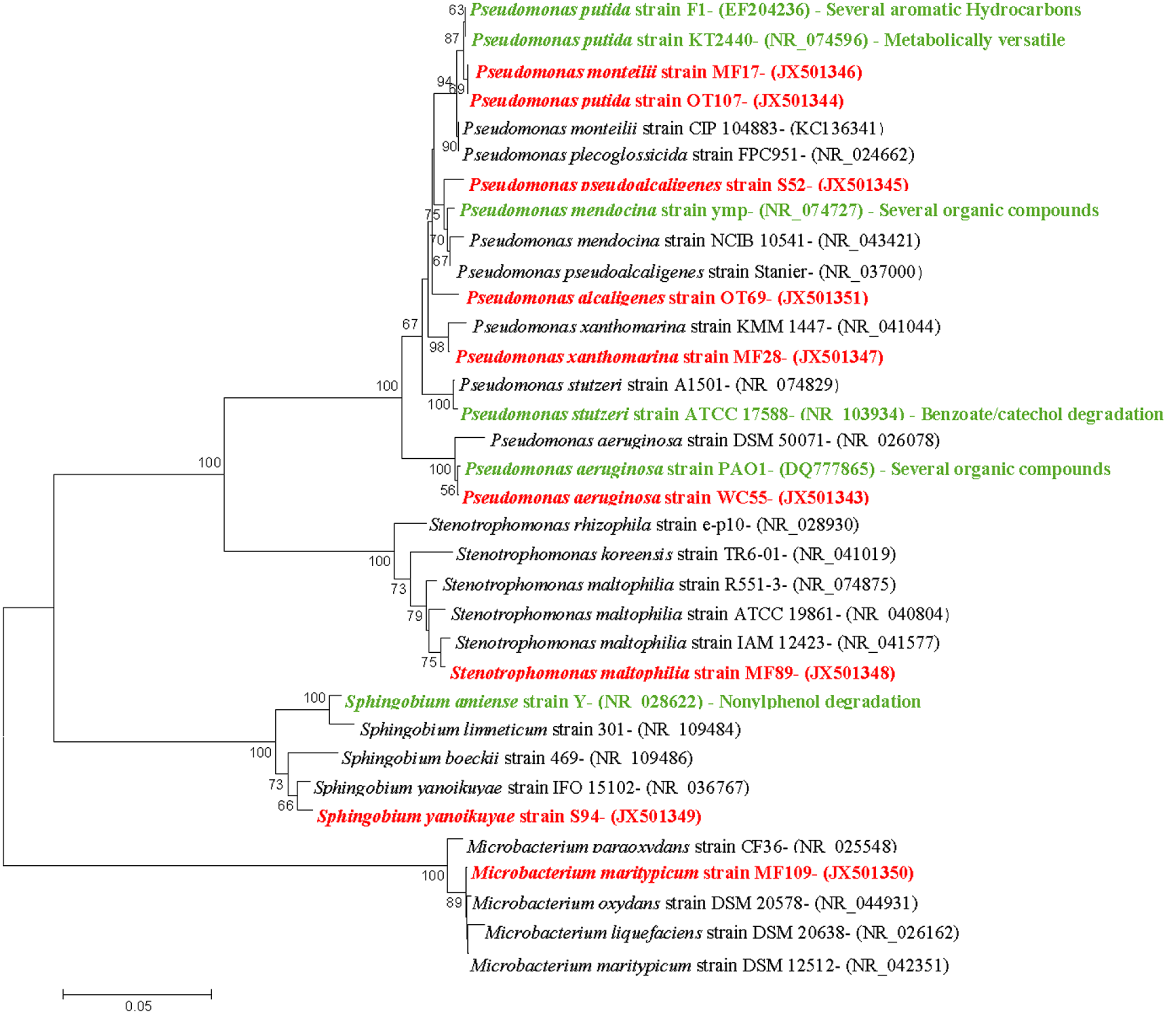
**Taxonomic affiliations of representative isolates shown as neighbor-joining dendrograms.** The optimal tree with the sum of branch length = 0.65751938 is shown. The percentage of replicate trees in which the associated taxa clustered together in the bootstrap test (500 replicates) are shown next to the branches. The evolutionary distances were computed using the Maximum Composite Likelihood method and are in the units of the number of base substitutions per site. The analysis involved 34 nucleotide sequences and positions containing gaps and missing data were eliminated. There were a total of 664 positions in the final dataset. The nine isolates obtained from oyster and its surrounding environments in this study are shown in red text; previously known biodegradative relatives are shown in green text.

Taxonomic affiliations of 72 pure bacterial strains isolated from the OT, MF, water, and S environments are summarized in **Tables 1** and **2**. We found a total of 10 different species across the oil-enriched samples with 80% of the isolated strains taxonomically affiliating to the *Pseudomonas* genus, as shown in **Figure 3**. This likely occurred because previous reports have shown that *Pseudomonas* predominates the oyster microbiome of both raw and retail oysters (Cao et al., 2009). Moreover, it is well-known that pseudomonads grow rapidly in the presence of high concentrations of nutrients (Greene et al., 2000; Rodríguez-Verdugo et al., 2012), such as those utilized in standard batch enrichments and hence, pseudomonads can easily outcompete other slow-growing bacteria that typically have lower Ks (Monod growth coefficient) and low maximum growth rates.

**Table 1:** Summary of oil-degrading bacterial strains isolated from DWH oil-amended microcosms containing oyster tissue (OT), mantle fluid (MF), water column (WC), and sediment (S) samples obtained from Dry Bar, Apalachicola Bay, FL, USA.

Isolated strains along with their original environment	NCBI match (% identity with nearest neighbor)	Bacterial division of the isolated strains	Accession number of strains chosen for oil degradation assay
Water column: 10^*^, 26^*^, 29^*^, 40^*^,41, 44^*^, 45^*^, **55**, 57^*^, 58, 64^*^, 67^*^, 68, 73, 74^*^, 75^*^, 76^*^, 82^*^, 92^*^, 99^*^, 100^*^, 110^*^, 115^*^ Oyster tissue: 48^*^, 59^*^	*Pseudomonas aeruginosa* strain PAO1 (99%)/*P. otitidis* strain MCC10330 (98%)	γ-Proteobacteria	JX501343
Sediment: 1, 3^¥^, 46, 47^¥^, 107^¥^, 108^¥^ Oyster tissue: 21^¥^, 88^¥^ Mantle fluid: 4, 5^¥^, 6^¥^, 8^¥^, 9^¥^, 11^¥^, 15, 16^¥^, **17**, 18^¥^, 19^¥^, 20, 27^¥^, 54, 56^¥^, 77, 78^¥^, 79, 87, 101^¥^, 105, 106	*P. monteilii* strain CIP 104883 (99%)/*P. plecoglossicida* strain FPC951 (99%)	γ-Proteobacteria	JX501346
Oyster tissue: **69**, 104, 116	*P. alcaligenes*	γ-Proteobacteria	JX501351
Sediment: 51, **52**, 53	*P. pseudoalcaligenes* strain Stanier 63 (98%)	γ-Proteobacteria	JX501345
Mantle fluid: **28**	*P. xanthomarina* strain KMM 1447 (99%)/*P. stutzeri* strain A1501 (99%)	γ-Proteobacteria	JX501347
Oyster tissue: 63, **107**	*P. putida* strain F1 (99%)	γ-Proteobacteria	JX501344
Mantle fluid: **89**, 91, 111, 113, 114	*Stenotrophomonas maltophilia* strain IAM 12423 (99%)	γ-Proteobacteria	JX501348
Sediment: **94**	*Sphingobium yanoikuyae* strain IFO 15102 (99%)	α-Proteobacteria	JX501349
Mantle fluid: **109**	*Microbacterium maritypicum* strain MF-C01 (100%)	Actinobacteria	JX501350

* Denotes strains that taxonomically affiliated with *P. otitidis.*

¥ ¥Denotes strains that taxonomically affiliated with* P. plecoglossicida, respectively.*

*Strains that appear in bold and underlined text were the ones that were selected for comparing their crude oil degradation propensities. Accession numbers of these nine strains appear in the last column.*

**Table 2:** Taxonomic affiliation shown of the 72 oil-degrading bacterial strains that were isolated from DWH oil-amended microcosms containing oyster tissue (OT), mantle fluid (MF), water column (WC), and sediment (S) samples obtained from Dry Bar, Apalachicola Bay, FL, USA.

Isolated strain affiliation	Oyster tissue	Mantle fluid	Water column	Sediment	Total isolated strains
*Pseudomonas monteilii*	2	22	–	6	30
*P. xanthomarina*	–	1	–	–	1
*P. pseudoalcaligenes*	–	–	–	3	3
*P. aeruginosa*	2	–	23	–	25
*P. alcaligenes*	3	–	–	–	3
*Stenotrophomonas maltophilia*	–	6	–	–	6
*Sphingobium yanoikuyae*	–	–	–	1	1
*P. putida *	2	–	–	–	2
*Microbacterium maritypicum*	–	1	–	–	1
Total sequences analyzed	9	30	23	10	72

Our findings are consistent with several other studies showing that bacteria from the Pseudomonadaceae family respond to degrade the DWH oil. In fact, a group studying the succession of hydrocarbon-degrading bacteria in the aftermath of the DWH oil spill recently reported that unmitigated oil from the wellhead early on in the spill resulted in the highest proportions of n-alkanes and cycloalkanes that corresponded with the predominance of Oceanospirillaceae and *Pseudomonas* (Dubinsky et al., 2013). In another study, 24 bacterial strains isolated from oiled beach sands were shown to comprise of 14 genera but all belonged to Gammaproteobacteria, including the well-known oil degrading bacteria- *Pseudomonas, Alcanivorax*, *Marinobacter*, and *Acinetobacter*, respectively (Kostka et al., 2011). Another group investigating the impact of DWH oil spill on microbial communities in wetland S and seawater samples collected along the Gulf shore recently reported that pyrosequencing based analysis showed 17 bacterial genera with the ability to degrade hydrocarbons, including- *Mycobacterium*, *Novosphingobium*, *Parvibaculum*, *Pseudomonas*, and *Sphingomonas*, in the contaminated S sample (Looper et al., 2013). Thus it appears that the Pseudomonads are a dominant community of hydrocarbon degrading bacteria in the Gulf of Mexico ecosystem, including the oyster microbiome, as shown by this study.

Further taxonomic characterization of the isolated strains revealed that all of WC isolates were ≥98% similar to either the *P. aeruginosa* or *P. otitidis group*, which is a closely related clade in the *Pseudomonas* family (Clark et al., 2006). Isolates from the OTs included *P. alcaligenes*, which are best known for degrading a variety of polycyclic aromatic hydrocarbon (PAH; Peix et al., 2009). In addition, OTs also contained isolates that were ≥98% similar to *P.putida*, as well as two isolates, *P. monteilii* and *P. plecoglossicida* that have been placed in the *P. putida* group (Peix et al., 2009); **Figure 3**. Isolates from the MF were found to be ≥98% similar to the *P. monteilii*, *P. plecoglossicida* group, however, MF also contained several other bacteria that were not found in the other environments, for example, *P. stutzeri* isolate MF28, which was ≥98% similar to *P. xanthomarina*, a novel bacteria that was originally isolated from marine ascidians (Romanenko et al., 2005). The MF also contained isolates that were ≥98% to *Stenotrophomonas maltophilia*, a common aquatic bacterium (Denton and Kerr, 1998) as well as an actinomycete – *Microbacterium maritypicum* (Takeuchi and Hatano, 1998; Schippers et al., 2005; **Table 1**; **Figure 3**). The S samples led to the isolation of two isolates that were also found in the OT and MF; these displayed ≥98% similarity to *P. plecoglossicida* and *P. monteilii*, respectively. However, the S also contained two isolates not found in any of the other surveyed environments- *P. pseudoalcaligenes*, a bacterium in the *P. aeruginosa* group, and *Sphingobium yanoikuyae*; an α-proteobacteria, capable of degrading a range of PAHs (Peix et al., 2009; Schuler et al., 2009).

**FIGURE 4:**
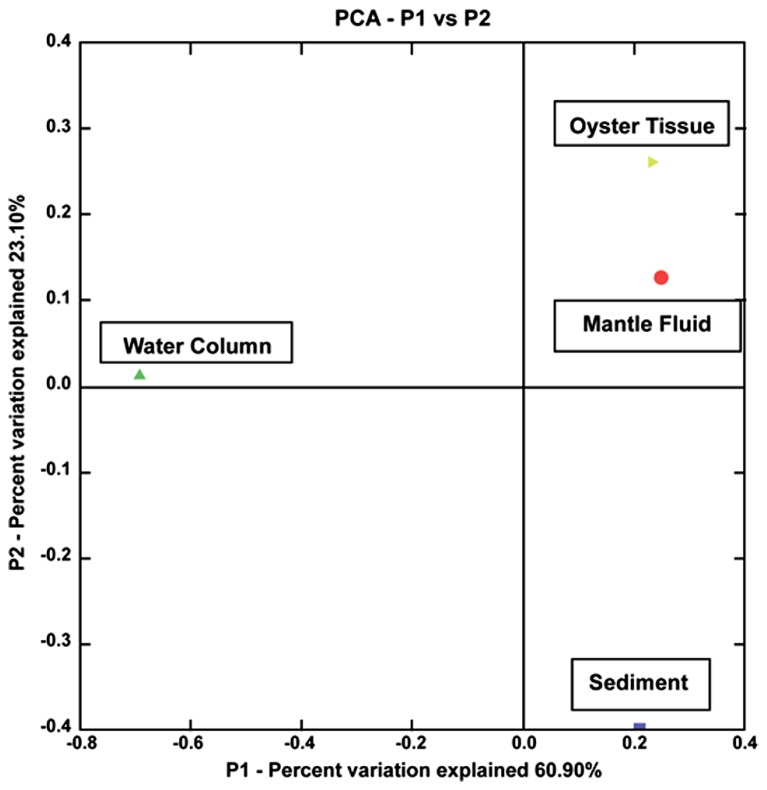
**Shown is a principal component analysis (PCA) obtained from UniFrac analysis of the 16S rRNA sequences of 72 isolated strains from the oyster tissue (yellow triangle), mantle fluid (red circle), water column (green triangle), and sediment (blue square).** The percentages on each of the axes show the percent variation explained by each axes.

To compare the 72 bacterial strains isolated from the WC, MF, OT, and S, a UniFrac based analysis was performed using the 16S rRNA sequences obtained in this study (Lozupone et al., 2006). As shown in **Figure 4**, results showed that the oil-degrading bacteria isolated from the OT and MF clustered strongly together on the same axis, however, S and WC bacteria separated out onto different axes. PCA axis 1 accounted for 60.90% of the variability, while PCA axis 2 accounted for 23.10%; together explaining 84% of the variability. Moreover, this data is in line with the ARISA findings, as the OT and MF bacteria in both the enriched samples and the isolated representatives are more similar than those that were present in the overlaying water or the S beds, suggesting the uniqueness of the oyster microbiome. King et al. (2012) recently showed that significant differences exist between stomach and gut microbiomes of the Eastern oyster, *Crassostrea virginica*. However, this and previous studies on the oyster microbiome have not compared the oyster-associated bacterial communities with those of the adjacent WC or S-environments on which the oysters are entirely dependent for their nutrition. Our study, thus, is unique because it provides a comparison of the oyster microbiome to those present in the surrounding oyster reef environment.

### GROWTH KINETICS OF THE OIL-DEGRADING BACTERIAL ISOLATES

**FIGURE 5:**
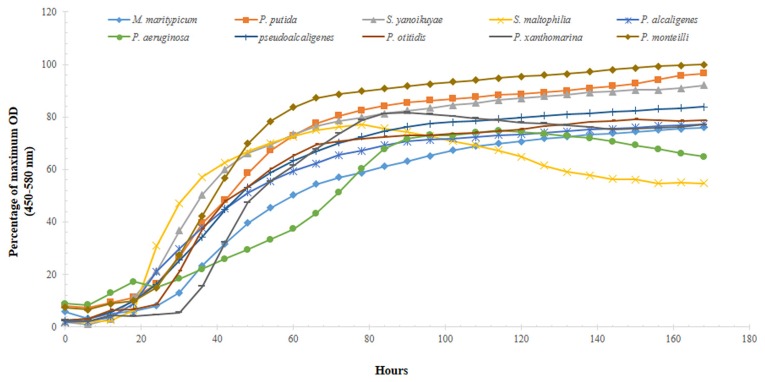
**Growth profiles of 10 selected isolated strains obtained from microcosms spiked with 0.75% DWH crude oil as the sole source of carbon and energy over the course of 7 days**.

Each of the nine isolates showed good growth over time in both, BH media containing 0.75% glucose or crude oil-spiked microcosms (**Figure 5**); the maximum optical density and doubling times of the isolates during the growth experiments are summarized in **Table 3**. Specifically, the two strains isolated from the OT and MF – *P. alcaligenes* OT69 and *P. xanthomarina* MF28, showed the fastest growth rates, with doubling times between 4.7 and 4.8 h, respectively (**Table 3**). This is most likely because the resident environment of these isolates provides a rich food supply, which facilitates their rapid growth and survival. Conversely, as is expected, the *P. aeruginosa* strain 55 isolated from the oligotrophic WC displayed the slowest doubling time (approximately 22 h), substantially longer than the other isolates (**Figure 5**). It appears that some of the isolated strains possess good catabolic potentials as compared with others reported previously; for example, Subathra et al. (2013) isolated 113 crude oil degrading S bacteria out of which 15 isolates were grown in BH media with 1% crude oil; gravimetric biodegradation loss was found highest at 55% by *P. aeruginosa* I5 isolate after 60 days of incubation. As compared to the previously isolated strain of *P. aeruginosa* I5, the *P. aeruginosa* srain WC55 isolated in this study was found to degrade 42% of Gulf crude oil in just over 7 days (see below), indicating its potent catabolic potential.

**Table 3:** Growth kinetics and oil degradation abilities of nine selected strains isolated from oyster tissue (OT), mantle fluid (MF), water column (WC), and sediment (S) samples obtained from Dry Bar, Apalachicola Bay, FL, USA.

Isolated strains	Maximum OD_450-580_	Average doubling time (h)	Oil loss (g)	Bacterial oil consumption (%)
Control	NA	NA	26.8 ± 0.6	NA
*P. monteilii* (MF17)	1.2 ± 0.02	8.0 ± 1.3	59 ± 1.6	32.2 ± 1.7
*P. xanthomarina* (MF28)	1.0 ± 0.01	4.7 ± 0.8	64.1 ± 4.0	37.2 ± 4.07
*P. pseudoalcaligenes* (S52)	1.0 ± 0.01	8.7 ± 0.5	64.9 ± 10.9	38 ± 10.9
*P. aeruginosa* (W55)	0.9 ± 0.04	21.9 ± 0.4	69.6 ± 2.7	42.7 ± 2.7
*P. alcaligenes* (OT69)	0.9 ± 0.03	4.8 ± 0.1	58.1 ± 10.6	31.2 ± 10.7
*Stenotrophomonas maltophilia* (MF89)	0.9 ± 0.05	5.5 ± 3.7	64.7 ± 3.1	37.8 ± 3.2
*Sphingobium yanoikuyae* (S94)	1.1 ± 0.01	4.6 ± 1.7	38.9 ± 8.6	12.0 ± 8.6
*P. putida* (OT107)	1.1 ± 0.06	9.5 ± 1.1	40.2 ± 9.6	13.3 ± 9.6
*Microbacterium maritypicum* (MF109)	0.9 ± 0.07	7.8 ± 0.8	64.6 ± 1.5	37.8 ± 1.6

Moreover, the growth experiment data was analyzed by one-way ANOVA, which revealed that significant differences (*P* = 0.001) existed between the growth rate of water-associated bacteria and those isolated from the OT, S, and the MF. This again, most likely reflects to the rich array of nutrient sources that are typically encountered in S and the oyster biome.

### OIL-DEGRADATION ABILITY OF THE ISOLATED BACTERIA

The percentage of crude oil lost abiotically and via microbially mediated mineralization processes is summarized in **Table 3**. *P. aeruginosa* strain WC55, which displayed the slowest doubling time relative to the other isolated strains, showed the highest utilization of crude oil (~42%). It seems likely that this WC -associated bacteria preferentially utilizes hydrocarbon fractions that are most abundant in Gulf crude oil, such as aliphatics C6–C35 (Kostka et al., 2011). Thus, despite of its slow growth, it preferentially and rapidly metabolized the predominant DWH oil fractions. In fact, we recently reported the whole genome sequence of *P. aeruginosa* WC55 compared with other four isolates, namely, *P. alcaligenes* OT69, *P. stutzeri* MF28, *Stenotrophomonas maltophilia* MF89, and *Microbacterium maritypicum* MF109, which confirmed the presence of as many as 958 putative genes for xenobiotic degradation and metabolism in strain WC55, which explains its higher (42%) biodegradation rate of DWH oil relative to other isolates. The other sequenced strains were found to contain lower numbers of putative xenobiotic and metabolism genes, which suggests that *P. aeruginosa* strain WC55 along with *P. alcaligenes* OT69, isolated from the oyster reef WC and OT possess good catabolic versatility (Chauhan et al., 2013).

*P. pseudoalcaligenes,* isolated from the S, achieved slightly higher degradation than *P. aeruginosa* at approximately 45% (**Table 3**). Several other isolated strains such as, *P. otitidis, P. monteillii*, *P. alcalignes*, *Stenotrophomonas maltophilia*, *Microbacterium maritypicum*, and *P. xanthomarina* showed almost similar biodegradation potentials within the range of 32–40%. Surprisingly, under the assay conditions, *P. putida*, which is a well-characterized hydrocarbon-degrader, consumed on an average 50% less oil relative to the other isolates. These oil degradation rates, however, should be interpreted with caution because it remains to be shown whether the isolated oyster-associated microbiota degraded Gulf crude oil individually or that mineralization occurs through assemblages of biodegradative consortia.

## CONCLUSIONS AND ECOLOGICAL IMPACT OF THIS STUDY

Gulf of Mexico oysters are prized for their economic value. Not only are oysters a valuable commodity as seafood; but oyster reefs provide a variety of ecosystem services including critical habitats for commercial fisheries, water filtration, and removal of excess nutrients from estuarine environments (Raj, 2008; Roosi-Snook et al., 2010; Piehler and Smyth, 2011; Grabowski et al., 2012). The value of this habitat, however, can be greatly reduced by toxic oil discharges, such as those from the 2010 DWH oil spill blowout. Effective response to such large-scale contamination of the marine environments requires a rapid and precise assessment of disturbances caused to the ecosystem such that timely mitigation strategies can be implemented. In this regard, biodegradative bacteria, as those identified from this and previous studies can significantly contribute to degradation of oil contaminants and rehabilitation of the perturbed environments. For example, stimulation of biodegradative communities within an impacted oyster reef ecosystem might be a potential remediation strategy to enhance cleanup and restoration efforts. However, baseline analyses of the oyster microbiome, which remains largely under-studied, are needed to develop successful approaches to problems of contamination of oyster reefs by petroleum and other hydrocarbons.

## Conflict of Interest Statement

The authors declare that the research was conducted in the absence of any commercial or financial relationships that could be construed as a potential conflict of interest.

## Footnotes

1 http://www.st.nmfs.gov/

2 http://greengenes.lbl.gov/
